# Strongyle infections and parasitic control strategies in German horses ― a risk assessment

**DOI:** 10.1186/s12917-014-0262-z

**Published:** 2014-11-12

**Authors:** Stephanie Schneider, Kurt Pfister, Anne M Becher, Miriam C Scheuerle

**Affiliations:** Comparative Tropical Medicine and Parasitology, Faculty of Veterinary Medicine, Ludwig-Maximilians-Universität, Leopoldstr. 5, D-80802 Munich, Germany

**Keywords:** Parasite control, Strongyle, *S. vulgaris*, Germany, Larval culture, FEC, Diagnosis, Selective anthelmintic therapy, Equine

## Abstract

**Background:**

As a consequence of the increasing levels of anthelmintic resistance in cyathostomes, new strategies for equine parasite control are being implemented. To assess the potential risks of these, the occurrence of strongyles was evaluated in a group of 1887 horses. The distribution of fecal egg counts (FECs), the frequency of anthelmintic drug use, and the deworming intervals were also analyzed. Between June 2012 and May 2013, 1887 fecal samples from either selectively or strategically dewormed horses were collected at 195 horse farms all over Germany and analyzed quantitatively with a modified McMaster technique. All samples with FEC ≥20 eggs per gram (EPG) were subjected to coproculture to generate third-stage larvae (LIII) for species differentiation.

**Results:**

Egg counts were below the limit of detection (20 EPG) in 1046 (55.4%) samples and above it in 841 (44.6%) samples. *Strongylus vulgaris* larvae were identified in two of the 841 positive samples. Infections with cyathostomes were found on every farm. The most frequently applied anthelmintic was ivermectin (788/50.8%), followed by pyrantel (336/21.6%). The mean time since last treatment was 6.3 months. High-egg-shedding (>500 EPG) strategically dewormed horses (183/1357) were treated, on average, three times/year. The planned treatment date was already exceeded by 72.5% of the high egg-shedders and by 58.1% of the moderate (200–500 EPG) and low egg-shedders (20–199 EPG).

**Conclusions:**

*S. vulgaris* seems to be rare in Germany and no difference in its frequency has yet been found between selectively treated horses and horses receiving treatment in strategic intervals. However, inconsistent parasite control has been observed. Therefore, to minimize the risks for disease, consistent and efficient parasite control should be implemented.

## Background

The spread of anthelmintic resistance in cyathostome parasites is of growing concern to the equine industry. The anthelmintic resistance of cyathostomes to benzimidazoles has been reported worldwide reviewed by [1]. Cases of pyrantel resistance in cyathostomines have also been reported in studies from various countries, including England, the United States, Italy, Brazil, Sweden, and Finland [1-8]. Several studies have also reported the reduced efficacy of ivermectin and moxidectin against small strongyles [9-12], and a reduction in the egg reappearance period (ERP) after treatment with ivermectin or moxidectin [12-16]. Furthermore, it is unclear whether new classes of drug will be developed in the near future [17].

It is now scientifically accepted internationally that the traditional approach to parasite control with frequent group-wise anthelmintic treatments at regular intervals (the so-called “strategic interval treatment”) has contributed to the development of resistance [1,2,18-20]. As a consequence and based on experience with alternative and targeted treatment schemes in ruminants reviewed by [21], a new strategy for equine parasite control was recently introduced to delay the development of anthelmintic resistance [22-25]. Selective anthelmintic therapy (SAT) is based on the selective treatment of horses with a fecal egg count (FEC) above a certain threshold [23,24,26,27]. Treatment thresholds of >200 eggs per gram (EPG) are often used and are accepted internationally [24,27,28]. The major aim of this strategy is to minimize the number of preventive treatments with anthelmintics to reduce the risk of the further development of resistance [24,28,29]. Horse owners in countries including Denmark, Sweden, the Netherlands, and Finland have widely and successfully used SAT for several years [23,29], and selective treatment schemes are also used in some horse farms in South Africa and England [22,27,30]. In Germany, the SAT method has been introduced in some areas and has led to a reduction in the frequency of anthelmintic treatments on these farms [24,25,31]. SAT also contributes considerably to the maintenance of a parasite refugium [32].

However, it is contentious whether reducing the frequency of treatments with the use of SAT will increase the likelihood that *Strongylus vulgaris* will complete its life cycle [24,28,33,34].

To assess the risks entailed in changing the anthelmintic treatment schemes, we evaluated the occurrence of strongyle infections (with an emphasis on *S. vulgaris*) in groups of German horses, including selectively treated horses and horses which were been treated in strategic intervals. The objectives of this study were to analyze: (a) the strongyle populations, focusing on large strongyles (especially *S. vulgaris*); (b) the distributions of the FECs and egg-shedding patterns; and (c) the application frequencies of anthelmintic treatments (the last-administered anthelmintic drug, the interval between the last deworming and sampling).

## Methods

### Farms and horses

Between June 1, 2012, and May 31, 2013, 192 German horse farms with a total of 1887 horses were included in this study. Most horses lived in southern (909/1887, 48.2%) or western Germany (311/1887, 16.5%). The number of horses examined on each farm ranged from 1 to 110 (mean = 9.3, median = 3). Each horse owner signed a declaration of consent before entering the study and agreed to follow ethical standards. The study was conducted with strict adherence to a high standard in veterinary care. Fecal samples were collected from the ground. The horse owners were asked to send freshly dropped fecal samples from their horses as part of the routine diagnostic practice. The following data were collected for each horse: age, name of the last-administered anthelmintic drug, time of the last anthelmintic treatment, mode of treatment, and the number of treatments per year for horses receiving treatments in strategic intervals. The horses were further divided into two subgroups according to the treatment strategy of the farm.

The inclusion criterion for both groups was that the last anthelmintic treatment had been administered at least 56 days (8 weeks) before sampling. Hence, the specific ERPs of commonly applied anthelmintics [35] was taken into account, so potential false negative results resulting from previous treatments were excluded. An anthelmintic drug containing moxidectin (moxidectin plus praziquantel) was only administered to three of the horses examined. Nielsen et al. [35] cited an ERP of 10–12 weeks for moxidectin. However, these three horses had last been treated ≥37 weeks before sampling, and hence were included in the study.

1. Horses treated according to a selective treatment scheme
  This group included 530 horses from 86 farms that administered SAT. These farms regularly performed FECs as an indication to deworm (FEC treatment threshold: 200 EPG). The majority of these farms had started using SAT 1–2 years before the study (i.e., 345/65.1% of the selectively treated horses). The ages of the horses in this group ranged from 2 to 39 years (mean =13.5 ± 6.37, median =13.0).
2. Horses treated according to a strategic interval treatment scheme
  This group included horses that were treated according to a strategic interval treatment scheme, i.e., treatment at regular deworming intervals (2–6 times a year) without any previous coprological testing. This horse group is herein after referred to as strategic horses/strategic horse group.
  In this group, 1357 horses from 106 farms were included and the ages of the horses ranged from 1 to 36 years (mean =13.0 ± 6.90, median =13.0).
  The age distributions in the selectively and strategically treated groups did not differ significantly (p =0.138, Man–Whitney *U* test; see “Statistics”).

### Fecal samples

One single fresh fecal sample from each horse was collected from the ground by the owner. The samples were packed in plastic boxes or bags and shipped overnight to the parasitology laboratory, where they were kept refrigerated and processed within 2 days.

The fecal samples were analyzed quantitatively using a modified McMaster technique, with a sensitivity of 20 EPG.

The levels of FECs in all 1887 horses examined were classified according to the guidelines suggested by the American Association of Equine Practitioners (AAEP) for classifying horses: low (20–199 EPG), moderate (200–500 EPG), or high (>500 EPG) egg-shedders [35]. Egg-shedding in horses with FEC <20 EPG was classified as below the limit of detection (<20 EPG).

### Larval culture

Larval culture (modified according to Roberts and O’Sullivan [36]) was performed for all 841 (44.6%) fecal samples with an FEC >20 EPG. In brief, 10 g samples of feces were weighed, put into plastic boxes, and incubated for 2 weeks at room temperature. During this time, the samples were regularly checked for desiccation, moistened if necessary, and ventilated for 1 hour every day. After incubation, the infectious larvae (L3) were harvested after sedimentation for at least 12 hour at 10°C (in a beaker). An aliquot of 100 μl was obtained from the sediment (1000 μl) containing the accumulated larvae. All the third-stage larvae (L3) of small and large strongyles, second-stage larvae (L2), and free-living rhabditiform nematodes were identified and counted. The remaining sediment (900 μl) was then analyzed for large strongyles. The strongyles were taxonomically identified according to Bürger and Stoye [37]. All cultures were examined by the same person.

### Statistics

The data were analyzed using IBM® SPSS® Statistics 21 (IBM Corporation, Armonk, USA) and Microsoft Excel 2010 (Microsoft Corporation, Seattle, USA). The distributions of the FEC results were compared between the two groups of differently treated horses using the Mann–Whitney *U* test. An independent-samples *t* test was used to compare the mean number of months between the last deworming and sampling for the two treatment groups. All statistical tests were deemed to be significant at p <0.05. The high-egg-shedding horses (FEC >500 EPG) of the strategically treated group were also investigated for deworming frequency, i.e., number of treatments per year, the last treatment occasion, and the next planned treatment date. Therefore, the “planned days between treatments per year” was estimated for each horse by dividing 365 days by the factor “treatments per year”, and this was added to the reported date of the last treatment to calculate the “planned next treatment date”. Seasonal influences were not considered in this calculation.

## Results

### Larval cultures

*S. vulgaris* was detected in the larval cultures from 2/841 strongyle-positive samples, i.e., a single sample from each treatment group. Only one *S. vulgaris* larva was identified in each treatment groups.

Cyathostomine larvae were found in at least one sample on every farm (both subgroups), making the prevalence 100% at the farm level. The mean number of cyathostomine larvae per aliquot (100 μl =1 g of feces) was 292 (median, 101); the maximal number per aliquot was 5872. No small strongyles at all were found in four of 841 larval coprocultures.

### FECs

**Figure 1:**
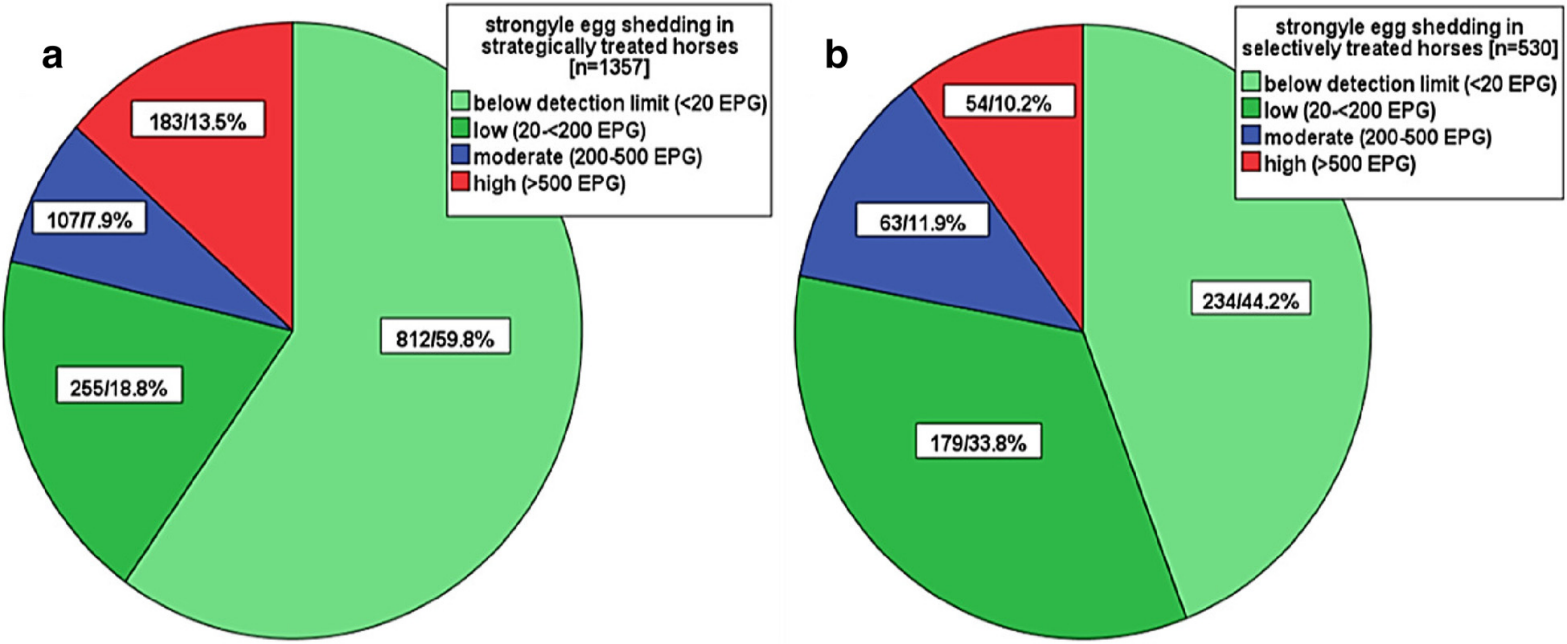
**Distributions of the egg-shedding levels.** Comparison of the selectively **(a)** and strategically **(b)** treated horse groups (numbers in each group and percentages).

Altogether, 1887 fecal samples were analyzed, and in 1046 (55.4%), FECs were below the level of detection. The distribution of FECs in all 1887 horses examined, separated into the strategic (1a) and selective groups (1b), is shown in Figure 1.

The FECs of the selectively treated group were distributed as follows: maximum = 4040 EPG, mean = 202 EPG, and median = 20 EPG. The FEC distribution for the strategically treated horses was: maximum = 8200 EPG, mean = 203 EPG, and median = 0 EPG. There was no statistically significant difference in the distributions of the FECs of the two treatment groups (Mann–Whitney *U* test). According to this classification, a low level of egg-shedding was found in 18.8% (255) and 33.8% (173) of horses in the strategically and selectively treated group, respectively, whereas 7.9% (107) of strategically treated horses and 11.9% (63) of selectively treated horses showed moderate levels of egg-shedding. More horses in the strategic group (13.5%, 183/1357) showed a high level of egg-shedding than in the selective group (10.2%, 54/530) (Figure 1a). The added percentage of horses “below the detection limit” and with “low egg-shedding” of the two treatment groups is comparable (78%).

### Deworming interval

**Figure 2:**
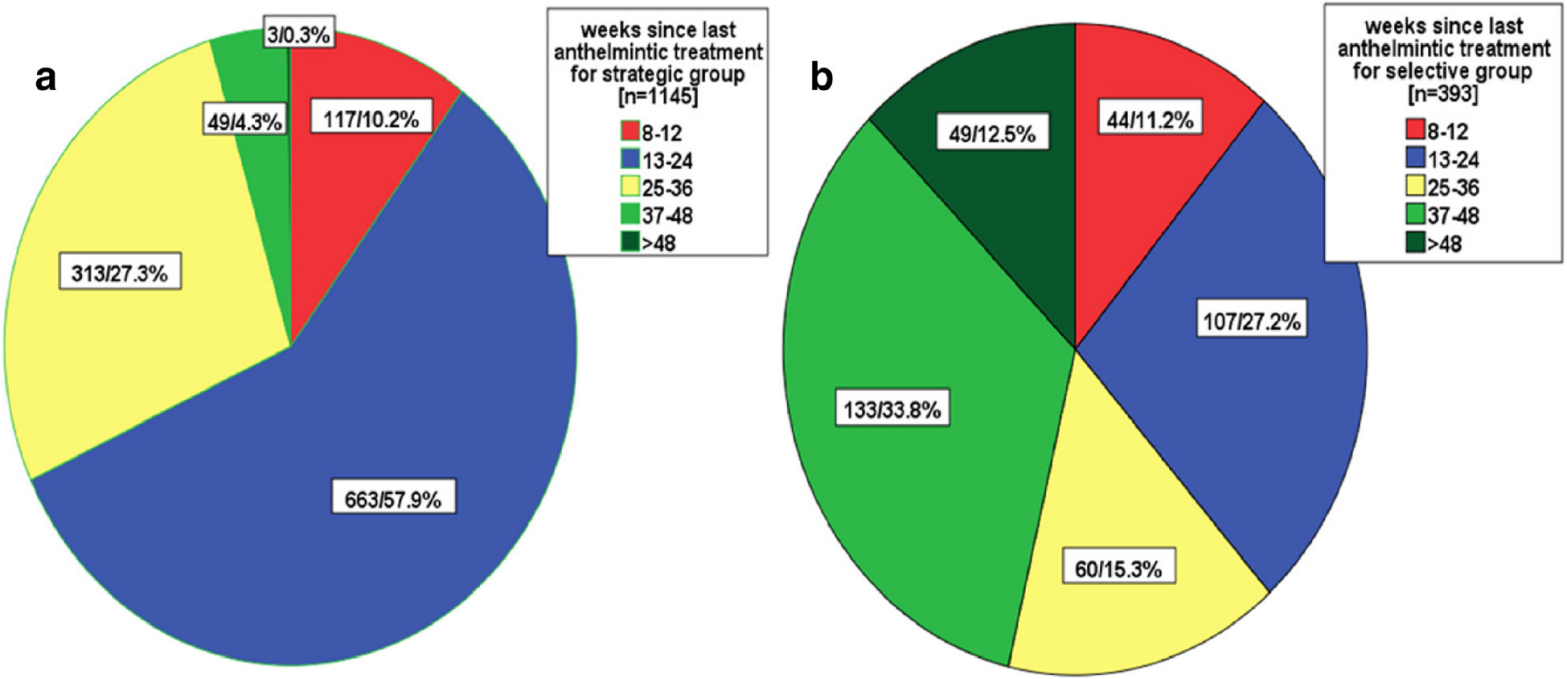
**Time (weeks) since the last anthelmintic treatment.** Comparison of the selectively **(a)** and strategically **(b)** treated horse groups (numbers in each group and percentages).

The data for 81.5% (1538/1887) of the horses were available for this analysis: 393 selectively treated horses and 1145 strategically treated horses. For all horses, the mean time between the last treatment and sampling was 6.3 months: selective group, 8.6 months; strategic group, 5.5 months. The difference in the mean number of months between the two groups was statistically significant (p = 0.000). The majority of strategically treated horses were dewormed less than 24 weeks before examination: 10.2% (117/1145) between 8–12 weeks and 57.9% (663/1145) between 13–24 weeks (Figure 2). In the strategic group, 27.3% (313/1145) of horses were dewormed 25–36 weeks before examination. In contrast, the horses in the selective group were dewormed less frequently. More than 50% of them were dewormed >24 weeks before sampling, 15.3% (60/393) 25–36 weeks before sampling, 33.8% (133/393) 37–48 weeks before sampling, and 12.5% (49/393) >48 weeks before sampling. Only 4.5% (52/1145) of strategically treated horses were dewormed >36 weeks before sampling: 49 horses 37–48 weeks before sampling and three horses >48 weeks before sampling (Figure 2).

### Last anthelmintic drug used

**Figure 3:**
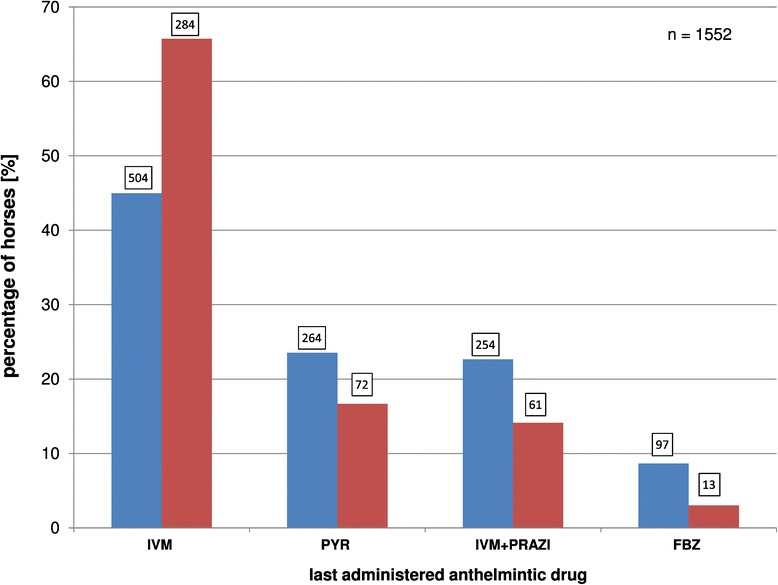
**Distributions (%) of the last-administered anthelmintic drugs among the horses (numbers in boxes).** Comparison of the selectively (red) and strategically (blue) treated horse groups. (FBZ, fenbendazole; IVM, ivermectin; PRAZ, praziquantel; MOX, moxidectin; PYR, pyrantel).

Data for 82.2% (1552/1887) of the horses were available for this analysis: 431 selectively treated horses and 1121 strategically treated horses. The distribution (%) of the last-administered anthelmintic drugs among the participating horses (numbers in boxes) is shown in Figure 3. Horse owners most frequently administered ivermectin (selective group, 65.7% [284/431]; strategic group, 45.0% [504/1121]). Pyrantel was administered to 16.7% (72/431) of the selectively treated horses and 23.6% (264/1121) of the strategically treated horses. The combination of ivermectin/praziquantel was used in 14.1% (61/431) of the selectively treated horses and 22.7% (254/1121) of the strategically treated horses. Three horses treated with the combination moxidectin/praziquantel (one selectively and two strategically treated horses) are not included in this figure.

### Distribution of FECs after deworming

**Figure 4:**
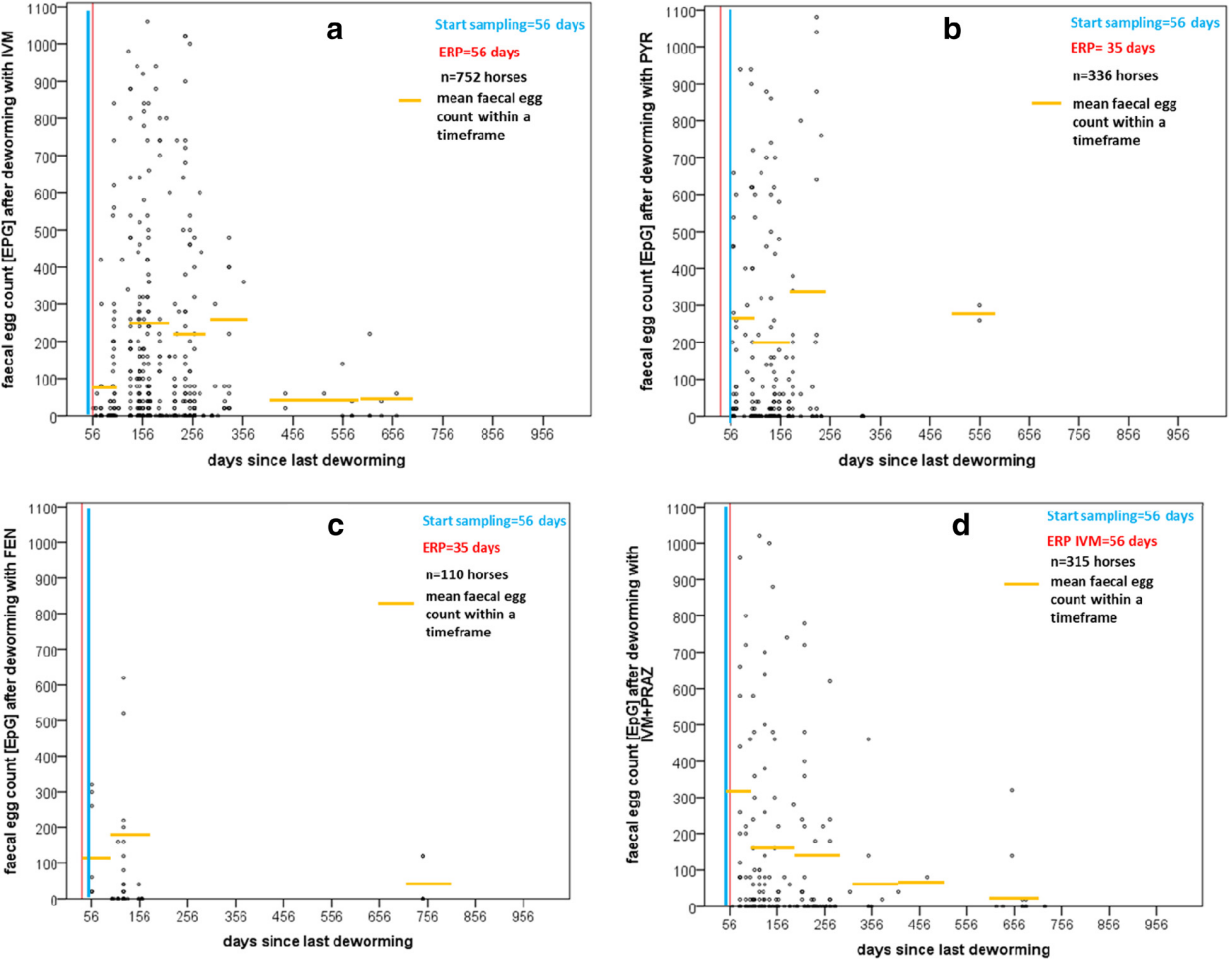
**Distributions of fecal egg counts (FECs) after treatment. (a)** With ivermectin (IVM); **(b)** pyrantel (PYR); **(c)** fenbedazole (FEN); and **(d)** ivermectin/praziquantel (PRAZ).

The distribution of FECs and the days between deworming with ivermectin (IVM, a), pyrantel (PYR, b), fenbendazole (FEN, c), or ivermectin/praziquantel (IVM/PRAZ, d) and sampling are presented in Figure 4. The corresponding ERPs, depending on which anthelmintic drug was used, are marked with red lines, and the mean FECs within the respective time frames (segment lengths: 100 days each) are marked with orange lines. The starting point for sampling was 56 days after the last deworming and is marked with a blue line.

Immediately after ERP, low-, moderate-, and high-egg-shedding horses were found in all four treatment groups (Figure 4). This was also true of horses that were dewormed >100 days before sampling. Zero EPGs were detected up to 746 days after treatment. Neither the individual FECs nor the mean FECs within the respective time frames (orange lines) increased with increasing time since the last treatment, regardless of the drug used (Figure 4).

### Deworming frequency and actual planned next treatment date

The deworming frequencies and the last and next planned treatment dates were examined in the high egg-shedders (183/1357, 13.5% of horses) in the strategic group. On average, three treatments per year (range: 2–6/year) were planned. The majority of horse owners planned two (78/183, 42.6%) or four (62/183, 33.8%) treatments per year. Three and six anthelmintic treatments were planned for 15.9% (29/183) and 1.1% (2/183) of strategically treated horses, respectively. No data for deworming frequency were received for 6.6% (12/183) of the strategically treated high-egg-shedding horses. When the estimated planned next treatment date was compared with the sampling date, 72.5% (124/171) of the strategically treated high egg-shedders had already exceeded their planned next treatment date, on average by 46 days.

The same estimations of the strategically treated moderate and low egg-shedders showed that 58.1% (187/322) of the horses (40 horses lacked the relevant data) were scheduled to be treated, on average, 40 days before the respective sampling date.

## Discussion

The spread of SAT and the first experiences of its implementation in the field have prompted discussion of the risks this treatment scheme might entail [29]. One major point of debate is the possible reemergence of *S. vulgaris*, potentially caused by a reduction in the frequency of anthelmintic treatments when SAT is administered [28,38,39]. Other potential problems in parasite control are the risk of the inappropriate implementation of the parasitic treatment scheme [39] and the failure to reliably identify high-egg-shedding horses, which are a higher contamination risk for other horses (because of the phenomenon of “egg-shedding consistency”) [24,26,32,40,41].

The risk of infection with *S. vulgaris* in Germany seems very low at the moment, because the low detection frequency of *S. vulgaris* presented here (2/841 *S. vulgaris*-positive samples) is consistent with results of several other studies in southern Germany, Brandenburg, and north Rhine-Westphalia [31,42-44]. Therefore, the situation in Germany seems to be very favorable for the implementation of SAT. In several neighboring countries, including Denmark, Poland, Italy, Switzerland, and the Netherlands, the prevalence of *S. vulgaris* is higher than that observed in Germany [38,40,45-49]. However, it must be kept in mind that the diagnostic and evaluation methods used in all these studies differed, which makes it difficult to interpret and compare the data. In the present study, individual fecal samples and the complete sedimented larvae from each horse were analyzed. In contrast, many protocols in other studies used pooled fecal samples [40,43,44,48,49], only identified the first 100 larvae [40,43,44,48], or only analyzed an aliquot of each sample [31]. The higher numbers of *S. vulgaris* stages reported in Poland and Italy can be attributed to the performance of postmortem analyses [45-47]. However, the Danish results [38] are more difficult to explain. They may be attributable to the comparatively high prevalence of *S. vulgaris* in Denmark in the past [50] and the long treatment intervals common there now [38] because a law introduced in 1999 requires that anthelmintic drugs be obtained on prescription [19]. Nielsen et al. [38] showed a mean time since the last deworming of 9.6 months, which is more than three months longer than that in the present study. This shows that SAT regimes may differ considerably, and the impact that these variations in the implementation of SAT will have in the long-term is unclear. The missing or irregular examination of larval cultures reportedly practiced by Danish veterinarians [28] might be another reason for the higher prevalence of *S. vulgaris* in that country. However, the prevalence of *S. vulgaris* must be monitored continuously, especially when trying to reduce the frequency of treatments [29]. There is a definite risk that *S. vulgaris* will spread in response to the higher *S. vulgaris* prevalence in neighboring countries. The strict implementation of SAT includes regular monitoring with FECs and larval cultures to analyze the FEC patterns and to limit the reemergence of large strongyles within a herd [29,33].

As expected, the ubiquitous occurrence of cyathostomes demonstrated by various German and international studies is supported by the findings of the present study [23,25,27,43,44,51-55]. Cyathostome larvae were found in at least one sample from every farm. This will not necessarily cause major problems, but heavy infections with cyathostomes must be prevented to avoid clinical larval cyathostominosis [56-58]. Therefore, treatment programs must be based on the control of cyathostomes.

The use of SAT (leaving animals untreated for some time) is made possible by the fact that the majority of horses are low egg-shedders [33,40,49,59,60], as observed in the present study. It is interesting to note that the added percentage of horses “below detection” and with “low egg-shedding” were almost equal (~78%) in both treatment groups. The fact that this is achievable with lower treatment frequencies in the selective group, supports the idea of SAT, providing that FEC <200 EPG are thought to be acceptable. The reported consistency of egg-shedding by individual horses over time [24,30,32,40] is an additional safety factor when the intervals between treatments or sampling are increased in stable groups of horses. However, the optimal intervals between fecal sampling must be determined individually [24,29]. Although long treatment intervals do not seem to affect most horses clinically, they may enable parasitic species with long life cycles, such as *S. vulgaris*, to spread and might lead to increasing infection pressure on the pasture [24,28,33,34]. Recent publications recommend at least one “safety” treatment for all horses in a herd once a year, together with strict quarantine measures for newly arrived horses [29,33,35].

Prolonged examination intervals in selectively treated horses, as well as undetected high egg-shedders, overdue treatments, and inappropriate treatment frequencies in strategically treated horses (as seen in the present study) may increase the risk of parasitic disease. More than half the high-egg-shedding strategically treated horses in this study were treated two or three times a year, which is insufficient to effectively reduce their egg-shedding rates. The insufficient involvement of veterinarians in parasite management programs might also explain the overdue treatments in the strategic horse group. Nevertheless, it has to be mentioned, that horse owners with good compliance and a chosen strategic treatment interval of 61 days (6 treatments/year), might easily be excluded due to the study design (no treatment with 56 days prior to sampling). However, recent German and international studies show that the majority of German horses are treated 2 to 4 times a year [39,61]. Therefore, we assume that a possible bias should be negligible. A positive side effect of SAT is that veterinarians will regain influence in parasite management programs, and regular examinations at the herd level will be practiced [19,28,33].

In this study, the selectively treated group showed lower FECs and contained fewer high egg-shedders than the strategically treated group. The risk of undetected high egg-shedders should also be lower in selectively treated horses than in strategically treated horses, if regular fecal sampling is reliably performed and if all horses that exceed the predetermined egg count threshold are dewormed [24,33,38].

As well as regular coproscopic diagnosis, the use of suitable, effective anthelmintics at adapted treatment intervals is important. As in other studies, ivermectin was the most widely used drug in this study [18,39,62,63]. Knowledge of the widespread benzimidazole resistance in cyathostomes reviewed by [1] and the efficacy of macrocyclic lactones (ivermectin and moxidectin) [4] might be responsible for this preference of ivermectin. However, the efficacy of any drug used must be verified at regular intervals [33,64,65].

In this study, only horses sampled after the respective ERP [35] were included, to eliminate false-negative FEC results caused by recent treatment. The FECs of all egg-shedding levels were observed immediately after ERP, regardless of the class of drug used. This supports the hypothesis of egg-shedding consistency, because the level of egg-shedding seems to be independent of the time since the last deworming, but is instead dependent on the status of the individual horse [24,30,32,40].

Every parasite control program should be based on continuous and consequent helminthological monitoring [20,33,66,67], the sensible use of anthelmintic drugs, including a “safety” treatment, or a few defined strategic interval treatments per year [29,33,68]. The efficacy of the anthelmintic drugs should also be monitored to detect resistance [2,33,64,65,68]. The AAEP recently presented guidelines for parasite control [35]. The introduction of similar practical guidelines for SAT and other new strategies could help veterinarians with the future implementation of effective parasite control.

## Conclusions

The majority of all horses examined were defined as low strongyle egg-shedders or below the limit of detection. Despite the long treatment intervals in some horses, no increase in FEC levels was found. However, some high egg-shedders remain undetected and without treatment for too long, especially in strategically treated groups. Our results also indicate that the risk of infections with large strongyles (*S. vulgaris*) in Germany is relatively small at the moment. To date, no effect of these two treatment strategies on the frequency of *S. vulgaris* has been determined. Thus, the implementation of SAT in Germany seems to be feasible at the moment. Nevertheless, a strict, regular, and consistent parasite management program, independent of the treatment strategy, is necessary for adequate and low-risk parasite control. Therefore, an internationally accepted guideline is urgently required to keep this risk low.
